# Tuberculose pulmonaire résistante à la rifampicine au Centre Hospitalier régional de Maradi, Niger (2014-2018)

**DOI:** 10.48327/mtsi.v3i4.2023.438

**Published:** 2023-11-06

**Authors:** Mahaman Laouali HAROUNA AMADOU, Ibrahim MAMAN LAWAN, Ousmane ABDOULAYE, Abdoul Kadir IBRAHIM MAMADOU, Oumarou AMADOU, Hassane BOUREIMA, Biraima AHAMADOU, Nouhou HAMA AGHALI, Nana Hadiza ABDOURAHAMANE MAIFADA, Abdoulaziz KABIROU AMOUSSA, Chaibou MAIDAKOUALE

**Affiliations:** 1 Service des maladies contagieuses, Université Dan Dicko Dankoulodo de Maradi, Centre hospitalier régional de Maradi, Niger; 2 Action Damien Maradi, Centre anti tuberculeux, Centre hospitalier régional de Maradi, Niger; 3 Service de biologie, Hôpital de référence de Maradi, Université Dan Dicko Dankoulodo de Maradi, Niger; 4 Centre hospitalier régional de Dosso, Dosso, Niger; 5 Service de médecine interne, Hôpital de référence de Maradi, Université Dan Dicko Dankoulodo de Maradi, Niger; 6 Service de CRENI/Pédiatrie, Centre hospitalier régional de Maradi, Niger; 7 Service de pneumologie, Centre hospitalier régional de Maradi, Niger

**Keywords:** Multidrug-resistant tuberculosis, HIV, Therapeutic failure, Treatment, Maradi, Niger, Sub-Saharan Africa

## Abstract

**Objectif:**

Décrire le profil clinique, thérapeutique et évolutif des patients suivis pour tuberculose résistante à la rifampicine (TB-RR) au Centre hospitalier régional (CHR) de Maradi (Niger) de 2014 à 2018.

**Méthodes:**

Nous avons mené une étude rétrospective et descriptive à partir des dossiers des patients suivis pour tuberculose multirésistante (TB-MR) entre le 1^er^ janvier 2014 et le 30 juin 2018 à l'unité de prise en charge de la tuberculose résistante de Maradi (Niger). Ont été retenus dans cette étude, les patients chez qui le diagnostic de TB-RR était posé.

**Résultats:**

Au total, 80 patients ont été inclus dans la présente étude (70 hommes et 10 femmes, âge moyen: 34,4 ans avec des extrêmes allant de 18 à 71 ans). La majorité des patients, 70%, avaient un IMC inférieur à 18 kg/m^2^. 7% des patients étaient séropositifs pour le VIH. Un patient était diabétique, 52% avaient des lésions radiologiques de degré 2. On notait une surdité de degré 1 au début du traitement chez 3% de nos patients et 96% étaient des cas précédemment traités pour la tuberculose. Un tiers des patients (36%) étaient des échecs de primo-traitement. Le protocole thérapeutique était le suivant: 4KmMfxPtoCfzHZE/5MfxCfzZE. Un seul patient avait une culture positive à la fin du 4^e^ mois de traitement. La plupart des patients avaient présenté des effets indésirables digestifs, surtout des vomissements. Le succès thérapeutique était de 88% avec 10% de décès, 1% d’échec thérapeutique et 1% de perdu de vue. Conclusion. Le régime thérapeutique court donne des résultats très satisfaisants avec des effets secondaires moindres.

**Motsclés:**

Tuberculose multirésistante, VIH, Échec thérapeutique, Traitement, Maradi, Niger, Afrique subsaharienne

## Abstract (en)

**Aim of study:**

To describe the clinical, therapeutic and evolutionary profile of patients followed for rifampicin-resistant pulmonary tuberculosis (RR-TB) at the Regional Hospital Centre (RHC) of Maradi (Niger) from 2014 to 2018.

**Methods:**

We conducted a retrospective and descriptive study based on the records of patients followed for Multidrug-resistant tuberculosis (MDR-TB) between January 1, 2014 and June 30, 2018 at the resistant tuberculosis management unit in Maradi (Niger). This unit is located within the RHC of Maradi and has a capacity of 20 beds in 4 wards. It receives patients with tuberculosis resistant to first-line anti-tuberculosis drugs. In this study, patients diagnosed with RR-TB by genotypic (GeneXpert MTB/RIF) or phenotypic (culture) testing were included. We excluded from this study: patients previously treated for more than 1 month with second-line anti-TB drugs; patients with resistance to second-line injectables (SLI) and/or fluoroquinolones (FQ); patients with an electrocardiogram QTc greater than 500 ms (the corrected QT (QTc) estimates the QT interval at a rate of 60 beats per second); cases of atypical mycobacteriosis detected by phenotypic testing.

Patients were previously on 2RHZE/4RH prior to the discovery of resistance. The treatment protocol for resistant TB was as follows: 4KmMfxPtoCfzHZE/5MfxCfzZE (The second-line injectable was replaced by Lzd in case of initial or treatment-emergent deafness). HIV co-infected patients received, in addition to anti-tuberculosis drugs, antiretrovirals and cotrimoxazole in preventive doses.

**Results:**

A total of 80 patients were included in the present study (70 males and 10 females, mean age 34.4 years with extremes ranging from 18 to 71 years). Patients aged 18-35 years accounted for more than half. Patients with primary treatment failure were the most frequent type (36%) followed by patients with retreatment failure (24%) and patients with retreatment relapse (17%). It should be noted that 77 patients (96%) were previously treated for TB and only 3 patients (4%) were new cases. The majority of patients (70%) had a Body mass index of less than 18 kg/m^2^. 7.5% of patients were HIV positive, one was diabetic, 52% of the patients had grade 2 radiological lesions. Grade 1 deafness was noted at the beginning of treatment in 3%. A third of patients (36%) were primary treatment failures. The treatment protocol was as follows: 4KmMfxPtoCfzHZE/5MfxCfzZE. Only 1 patient had a positive culture at the end of the 4th month of treatment. Most of the patients had experienced adverse events, mainly digestive, with vomiting being the most common. The therapeutic success rate was 88%. We noted 10% of deaths, 1% of therapeutic failure and 1% of lost to follow-up.

Six months after treatment, 48 patients (60%) were smear negative and 43 (54%) were culture negative. In 32 patients (40%), the smear was not performed and culture was not performed in 37.

**Conclusion:**

The short treatment regime gives satisfactory results in the absence of resistance to fluoroquinolones, with rare adverse effects. In Niger, further efforts should be made to minimize the delay in diagnosis which is responsible for most deaths during treatment. A centre could usefully be designated to organize “TB consiliums” allowing any practitioner to submit difficult cases of MDR-TB.

## Introduction

Selon l'OMS, la tuberculose (TB) chronique est un cas de tuberculose en échec au régime de retraitement, donné sous la supervision directe d'un agent de santé. Cette TB est due aux bacilles multirésistants, définis par la mise en évidence de bacilles résistants au moins à l'isoniazide et à la rifampicine [8].

La tuberculose constitue un véritable problème de santé publique à l’échelle mondiale. C'est l'une des 10 principales causes de décès à travers le monde et la première cause de décès dû à un seul agent infectieux. En 2017, le nombre de personnes ayant développé cette maladie a été estimé à 10 millions dont la majorité se trouvent en Asie et en Afrique subsaharienne [12].

La tuberculose résistante est un défi de santé publique. À travers le monde en 2017, 558 000 cas étaient estimés comme ayant une tuberculose résistante à la rifampicine (TB-RR), le plus puissant antituberculeux de première ligne, et parmi eux 82% avaient une tuberculose multirésistante (TB-MR) [12].

L'OMS estime qu'entre 36 000 et 44 000 cas de TB-MR sont survenus dans la Région Afrique en 2016. Parmi ces derniers, 15% ont développé une résistance à la rifampicine (le médicament de première intention le plus efficace) et ont nécessité un traitement contre la TB-MR [11].

La charge de la TB-MR incombe en grande partie à trois pays – la Chine, l'Inde et la Fédération de Russie – qui représentent ensemble près de la moitié des cas mondiaux. Environ 9,5% des cas de TB-MR étaient atteints de tuberculose ultrarésistante en 2015 [11].

À ce jour, tous les pays de la Région Afrique, à l'exception des Seychelles, ont notifié des cas de TB-MR et 13 pays ont déjà signalé des cas de tuberculose largement résistante aux médicaments (XDR-TB). Le sous-diagnostic des formes multirésistantes reste important, évalué par l'OMS à 68% en Afrique [11].

Au Niger, le traitement de la TB-MR a réellement débuté en 2008 et un cumul de 2 548 malades a été testé par des méthodes appropriées. Parmi ces derniers, 483 malades ont été confirmés résistants parmi lesquels 424 ont été mis sous régime court de 9 à 11 mois. Le taux de guérison enregistré pour la cohorte de 2017 est de 85,5% [5, 16].

L'objectif de notre étude était de:

- Décrire les caractéristiques sociodémographiques des patients traités au Centre hospitalier régional de Maradi (CHRM), Niger (2014-2018).
- Rapporter les effets indésirables liés aux molécules utilisées ainsi que leur degré de sévérité au CHRM (2014-2018).
- Identifier l'issue thérapeutique des patients au CHRM.

## Patients et méthodes

Nous avons mené une étude rétrospective et descriptive à partir des dossiers des patients suivis pour TB-MR entre le 1^er^ janvier 2014 et le 30 juin 2018 à l'unité de prise en charge de la tuberculose multirésistante de Maradi (Niger). Cette unité située dans l'enceinte du CHRM a une capacité d'accueil de 20 lits dans 4 salles. Elle reçoit les patients atteints de tuberculose résistante aux antituberculeux de première ligne.

Étaient inclus les patients ayant une TB-RR confirmée par un test génotypique (GeneX-pertMTB/RIF) ou phénotypique (la culture). Étaient exclus:

- Les patients antérieurement traités pendant plus de 1 mois par les antituberculeux de 2^e^ ligne.
- Les patients ayant une résistance aux injectables de seconde ligne (ISL) et/ou aux fluoroquinolones (FQ).
- Les patients ayant à l’électrocardiogramme un QT corrigé (QTc) supérieur à 500 ms. Le QTc estime l'intervalle QT à un rythme de 60 battements par seconde. Cela permet la comparaison de valeurs de QT à differents rythmes et améliore la détection de patients à risque d'arythmies en raison d'un intervalle QT prolongé.
- Les cas de mycobactériose atypique détectée au test phénotypique.

Les cas de tuberculose étaient définis comme suit:

- Nouveau cas (N): patient qui n'a jamais été traité pour une tuberculose ou qui n'a jamais pris d'antituberculeux pendant plus d’1 mois.
- Échec thérapeutique chez un nouveau cas (E1): patient dont le frottis demeure positif à 5 mois ou plus tard au cours de son primo-traitement.
- Échec thérapeutique chez un cas en retraitement (E2): patient dont le frottis demeure positif à 5 mois ou plus tard au cours de son retraitement.
- Rechute (R1 et R2): patient qui a été traité pour la tuberculose par un régime de primo-traitement (R1) ou retraitement (R2), a été déclaré « Guéri » ou « Traitement terminé » mais est à nouveau diagnostiqué tuberculeux avec une bactériologie (frottis ou culture) positive.
- Reprise de traitement (RT): patient à frottis positif qui reprend le traitement après une interruption de plus de 2 mois consécutifs (qui avait été déclaré « Perdu de vue »).

Le traitement de la TB-RR était constitué d'un régime thérapeutique court variant de 9 à 11 mois avec une phase intensive de 4 mois qui doit être prolongée jusqu'au 5^e^ mois si le frottis est positif au 4^e^ mois et jusqu'au 6^e^ mois si le frottis est positif au 5^e^ mois; cette phase était suivie d'une phase fixe de continuation de 5 mois. Le traitement directement observé était administré chaque jour par un agent de santé tout au long de la durée du traitement selon le protocole thérapeutique suivant :

4KmMfxPtoCfzHZE/5MfxCfzZE (Km: Kanamycine; Mfx: Moxifloxacine; Gfx: Gantifloxacine; Pto: Prothionamide; H: Isoniazide; Cfz: Clofazimine; E: Éthambutol; Z: Pyrazinamide; Lzd: Linézolide; ISL: Injectable de seconde ligne; 4 et 5 déterminent les mois). L'ISL était remplacé par le Lzd en cas de surdité initiale ou apparue au cours du traitement.

Tous ces patients étaient antérieurement mis sous antituberculeux selon le protocole national de lutte contre la tuberculose au Niger: la rifampicine (R), l'isoniazide (H), la pyrazinamide (Z) et l’éthambutol (E) étaient utilisés les 2 premiers mois puis la rifampicine et l'isoniazide pour les 4 mois suivants (2RHZE/4RH). La phase initiale intensive (2RHZE) commençait avec 4 comprimés le matin 45 à 60 minutes avant le premier repas. Les résultats du traitement étaient définis ainsi:

- Guérison – Patient atteint de TB pulmonaire avec une TB confirmée bactériologiquement au début du traitement qui a terminé son traitement tel que recommandé par la politique nationale, avec des preuves de réponse bactériologique et aucune preuve d’échec.
- Traitement terminé – Patient qui a terminé le traitement tel que recommandé par la politique nationale, dont le résultat ne répond pas à la définition de guérison ou d’échec du traitement.
- Décès – Patient décédé pour une raison quelconque avant ou au cours du traitement.
- Échec thérapeutique – Patient dont le schéma thérapeutique a dû être arrêté ou définitivement changé pour un nouveau régime ou stratégie de traitement pour une des raisons suivantes:
  1. aucune réponse clinique et/ou aucune réponse bactériologique;
  2. effets indésirables graves des médicaments;
  3. preuve d'une acquisition de résistance aux médicaments du régime.
- Perdu de vue – Patient dont le traitement a été interrompu pendant 2 mois consécutifs ou plus.
- Non évalué – Patient pour lequel aucun résultat de traitement n'est attribué (cela inclut les patients transférés vers une autre unité de traitement et dont le résultat de traitement est inconnu).
- Succès thérapeutique – Somme des patients guéris et des patients ayant terminé leur traitement.

Les patients co-infectés par le VIH recevaient, en plus des antituberculeux, des antirétroviraux et du cotrimoxazole à dose préventive. La première ligne thérapeutique antirétrovirale était constituée de ténofovir, de lamivudine et d’éfavirenz selon le protocole du Programme national de lutte contre le VIH au Niger.

Les données collectées étaient le profil sociodémographique du patient (âge, sexe, niveau d'instruction scolaire, profession, statut matrimonial, lieu de résidence); la clinique (antécédents médicaux liés ou non au traitement antituberculeux, forme clinique de la tuberculose, statut sérologique VIH, effets secondaires rapportés ou signalés par les patients); la bactériologie. Ces données étaient recueillies en utilisant les dossiers individuels des patients et le registre TB-RR du centre qui incluaient tous les paramètres que nous avons étudiés. Elles ont été analysées à l'aide du logiciel IBM-SPSS dans sa version 20.

## Résultats

Au total, 80 patients ont été inclus dans notre étude. Le Tableau I résume les caractéristiques sociodémographiques et le type des patients. L’âge des patients variait de 18 à 71 ans, avec une moyenne de 34,4 ans. Les 18 à 35 ans représentaient plus de la moitié des patients. Le sexe-ratio était de 7. Les patients en échec de primo-traitement étaient le type le plus fréquent (36%) suivi des patients en échec de retraitement (24%) et des patients en rechute de retraitement (17%). Il faut noter que 77 patients soit 96% étaient des cas précédemment traités pour la tuberculose et seulement 3 patients (4%) étaient de nouveaux cas. La majorité des patients (71%) avaient un IMC < 18 kg/m^2^. Six patients étaient positifs au VIH soit un taux de 7,5% et un seul était diabétique (1%). Les deux tiers (65%) présentaient un taux d'hémoglobine inférieur à 11 g/dL.

**Tableau I:** Caractéristiques selon le sexe, l’âge, les antécédents et l'IMC de 80 patients souffrant de tuberculose résistante à Maradi Characteristics by gender, age, case history and BMI of 80 patients with MDR tuberculosis in Maradi

Variables	Nombre (%)
Sexe	
hommes	70 (87)
femmes	10 (13)
sex-ratio	7
Âge (ans)	
moyenne	34,4
extrêmes	18 – 71
18 – 25	21(26)
26 – 35	31(39)
36 – 45	17 (21)
46 – 55	8 (10)
56 – 71	3 (4)
Antécédents	
échec primo-traitement	29(36)
échec de retraitement	19(24)
rechute de retraitement	14 (17)
rechute de primo-traitement	12 (15)
reprise de traitement	3 (4)
nouveau cas	3 (4)
IMC	
<18 kg/m^2^	57 (71)
19-25 kg/m^2^	23(29)

Respectivement 75 patients (94%) et 72 patients (90%) sont devenus négatifs au frottis et à la culture à la fin des 4^e^ et 6^e^ mois de traitement (Tableau II).

**Tableau II:** Caractéristiques biologiques et radiologiques de 80 patients souffrant de tuberculose résistante à Maradi Biological and radiological data of 80 patients with MDR tuberculosis in Maradi

Variables	Nombre (%)
Statut VIH	
positif	6 (7)
négatif	74 (93)
Terrain diabétique	
diabétique	1 (1)
non diabétique	79 (99)
Taux d'hémoglobine	
< 11 g/dL	52 (65)
12-15 g/dL	28 (35)
Frottis à la fin du 4e mois de traitement	
négatif	75 (94)
positif	1 (1)
patient décédé	4 (5)
Frottis à la fin du 6e mois de traitement	
négatif	72 (90)
positif	0
patient décédé	8 (10)
Lésions radiologiques	
degré 1	11(14)
degré 2	42 (52)
degré 3	27 (34)

Les patients ont présenté, à des degrés de sévérité variables, des effets indésirables digestifs, hépatiques, auditifs, cutanés, hématologiques et cardiaques (Tableau III). Ainsi, 69% ont présenté des effets indésirables digestifs à type de vomissements, 53% des effets secondaires hépatiques et/ou auditifs, 8% des effets indésirables cutanés et/ou hématologiques, 6% des effets indésirables cardiaques.

**Tableau III:** Effets secondaires observés en cours de traitement chez 80 patients souffrant de tuberculose résistante à Maradi Observed side effects during the treatment of 80 patients with MDR tuberculosis in Maradi

Variables	Nombre (%)
Effet secondaire digestif	
aucun	**26 (32)**
degré de sévérité 1	**29 (36)**
degré de sévérité 2	**25 (31)**
degré de sévérité 3	**0**
Effet secondaire hépatique	
aucun	**38 (47)**
degré de sévérité 1	**33 (41)**
degré de sévérité 2	**7 (9)**
degré de sévérité 3	**2 (3)**
Effet secondaire auditif	
aucun	**38 (47)**
degré de sévérité 1	**33 (41)**
degré de sévérité 2	**7 (9)**
degré de sévérité 3	**2 (3)**
Effet secondaire cutané	
aucun	**74 (92)**
degré de sévérité 1	**3 (4)**
degré de sévérité 2	**3 (4)**
degré de sévérité 3	**0**
Effet secondaire cardiaque	
aucun	**75 (94)**
degré de sévérité 1	**1 (1)**
degré de sévérité 2	**4 (5)**
degré de sévérité 3	**0**
Effet secondaire hématologique	
aucun	**74 (92)**
degré de sévérité 1	**3 (4)**
degré de sévérité 2	**3 (4)**
degré de sévérité 3	**0**

Le 4KmMfxPtoCfzHZE/5MfxCfzZE était le type de traitement le plus utilisé totalisant ainsi 76 patients (95%). Soixante-dix patients (87%) étaient guéris à la fin du traitement, 8 patients (10%) étaient décédés, 1 patient en échec et 1 perdu de vue.

Les résultats du frottis et de la culture 6 mois après traitement étaient: 48 patients (60%) ont négativé leur frottis et 43 (54%) ont négativé leur culture. Le frottis n'a pas été réalisé chez 32 patients (40%), et la culture chez 37 (46%) (Tableau IV).

**Tableau IV:** Traitements et résultats chez 80 patients souffrant de tuberculose résistante à Maradi Treatments and outcome for 80 patients with MDR tuberculosis in Maradi

Variables	Nombre (%)
Type du traitement	
4KmMfxPtoCfzHZE/5MfxCfzZE	**76 (95)**
4LzdMfxPtoCfzHZE/5MfxCfzZE	**4 (5)**
Résultats du traitement	
guéri	**70 (88)**
décédé	**8 (10)**
perdu de vue	**1 (1)**
échec	**1 (1)**
Résultat du frottis 6 mois après le traitement	
négatif	**48 (60)**
non réalisé	**32 (40)**
Résultat de la culture 6 mois après le traitement	
négatif	**43 (54)**
non réalisé	**37 (46)**

**Figure 1:**
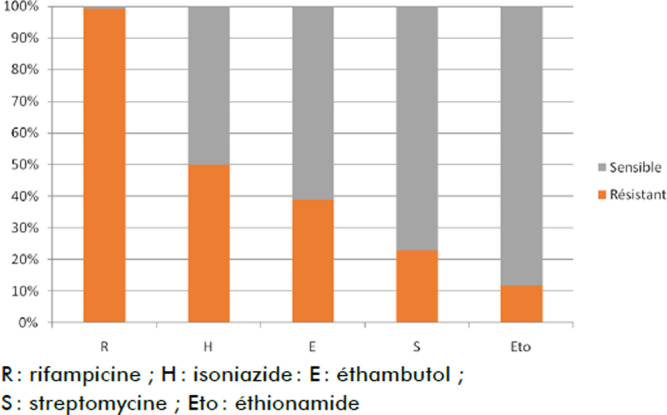
Tests de sensibilité chez 80 patients souffrant de tuberculose résistante à Maradi Sensitivity tests for 80 patients with MDR tuberculosis in Maradi

Le test de sensibilité est un examen clef pour la confirmation de la résistance aux antituberculeux. Selon le test de sensibilité pratiqué chez tous les patients, nous avons trouvé la résistance à R, H, S, E et Eto respectivement dans 100%, 50%, 42%, 28% et 10% des cas (Fig. 1).

## Discussion

Les objectifs de cette étude en termes de description des caractéristiques sociodémographiques, des effets indésirables liés aux molécules et d'identification de l'issue thérapeutique des patients ont été atteints. Cependant, comme toute étude rétrospective, celle-ci présente des données incomplètes que seule une étude prospective pourrait corriger en incluant un grand nombre de patients et en recueillant des données complètes. Cette étude pourrait concerner tous les centres de prise en charge de la tuberculose résistante au Niger.

La majorité des patients étaient des échecs de primo-traitement ou de retraitement. Une explication est que la plupart d'entre eux, quand ils commencent à se sentir mieux, n'attendent pas la fin du traitement pour se rendre dans les pays voisins. Face à ces situations d'abandon, des mesures doivent être prises pour permettre non seulement à ces malades de bien se traiter, mais aussi de ne pas propager cette maladie.

Notre étude a montré une prédominance masculine avec un sex-ratio égal à 7. Cette prédominance est observée dans la plupart des études menées à travers le monde [8, 13, 14]. Néanmoins, d'autres études avaient retrouvé une prédominance féminine [7, 11]. Cette prédominance du sexe masculin pourrait s'expliquer par les comportements à risque (alcoolisme, toxicomanie, tabagisme) plus souvent présents chez les hommes.

Dans notre étude, la moyenne d’âge des patients était de 34,4 ans. Plusieurs études ont trouvé des moyennes plus ou moins proches de la nôtre. Ainsi Piubello *et al.* [15] ont noté une moyenne d’âge de 31 ans, Kuaban *et al.* [10] de 34 ans, Kashongwe *et al.* [9] de 34 ans. Une moyenne d’âge plus élevée est notée par Camara *et al.* [4], de l'ordre de 43 ans.

La majorité des patients (71%) avait un IMC < 18 kg/m^2^. Cet état nutritionnel est observé dans plusieurs études à des degrés variables [10, 15]. Ceci s'explique par la présence d'un des signes d'imprégnation tuberculeuse que sont l'anorexie et d'autres facteurs favorisant la tuberculose comme la pauvreté, la promiscuité, l'immunodépression…

S'agissant de l'immunodépression, 6 patients étaient positifs au VIH soit un taux de 7,5% et 1 seul était diabétique. Ces deux pathologies sont à chercher dans la prise en charge effective de la tuberculose. Ouédraogo *et al.* [13] au Burkina Faso avaient trouvé un taux de séroprévalence égal au nôtre: 7,5%.

Globalement, le contrôle de la co-infection tuberculose-VIH devenait problématique, provoquant l'augmentation de la tuberculose multirésistante et ultrarésistante [12].

De nouvelles recommandations concernant le régime court de 9 mois pour le traitement de la tuberculose multirésistante ont été publiées par l'OMS en 2016. Ces recommandations remplacent les précédentes qui préconisaient un traitement d'une durée minimale de 20 mois avec au moins 8 mois d'injectables de seconde ligne. Ces recommandations se basaient sur les résultats préliminaires d’études parmi lesquelles celle menée au Bangladesh par Action Damien à partir des travaux pionniers du Dr Armand Van Deun, et celle coordonnée par l'Union Internationale contre la Tuberculose et les Maladies Respiratoires dans 9 pays d'Afrique francophone grâce au financement d'Expertise France. Ces études ont montré un très bon succès thérapeutique (> 80%) y compris chez les patients infectés par le VIH, avec des effets indésirables relativement limités [3]. Nos résultats, de l'ordre de 87% de réussite, sont en faveur du maintien du régime court pour traiter la tuberculose multirésistante.

Certains des effets indésirables liés au traitement sont notés chez quelques-uns de nos patients. Ils sont pour la plupart de faible sévérité. D'autres études avaient aussi retrouvé des effets indésirables liés aux médicaments [1, 2, 6]. La plupart de ces effets indésirables ont été observés au cours des 4 premiers mois du traitement. Ceci peut être dû au nombre de médicaments utilisés pendant la première phase (7 molécules). Ces effets diminuent avec le temps mais peuvent être sévères et nécessiter l'adjonction de traitements complémentaires symptomatiques, voire la modification du régime thérapeutique.

L'Action Damien et le Fonds mondial approvisionnent gratuitement nos centres en antituberculeux de deuxième ligne depuis le début de la prise en charge de la tuberculose résistante. Par conséquent, jusqu’à ce jour nous n'avons pas connu de rupture de stock. Pour pérenniser cette prise en charge de la tuberculose résistante, l’État du Niger envisage de trouver des mécanismes garantissant l'approvisionnement de nos differents centres de santé en antituberculeux comme pour le cas de la tuberculose sensible.

## Conclusion

La tuberculose pulmonaire résistante à la rifampicine progresse, compte tenu d'un certain nombre de facteurs dont le traitement antituberculeux mal conduit et le retard dans le diagnostic des patients. Cependant, le régime thérapeutique court donne des résultats très satisfaisants en l'absence de résistance aux fluoroquinolones et avec des effets indésirables moindres qu'un régime long. Au Niger, des efforts supplémentaires sont à faire en vue de minimiser le retard diagnostic responsable de la plupart des décès au cours du traitement. Un centre pourrait être utilement désigné pour organiser des « TB consiliums » permettant à tout praticien de soumettre ses cas difficiles de tuberculose multirésistante. Ce centre devrait bénéficier du matériel permettant d’étudier les souches multirésistantes (résistances supplémentaires aux fluoroquinolones, à la kanamycine…).

## Contribution des auteurs

Mahaman Laouali HAROUNA AMADOU: rédacteur principal du manuscrit, concepteur et correspondant de l’étude.

Ousmane ABDOULAYE: a participé à la recherche bibliographique de l'article.

Ibrahim MAMAN LAWAN: a participé à la collecte et au traitement des données.

Abdoul Kadir IBRAHIM MAMADOU: corédacteur du manuscrit.

Oumarou AMADOU: a participé à la collecte des données.

Hassane BOUREIMA: a participé à la collecte des données.

Biraima AHAMADOU: a participé à la collecte des données.

Nouhou HAMA AGHALI: a participé à la recherche bibliographique de l'article.

Nana Hadiza ABDOURAHAMANE MAIFADA: co-rédactrice du manuscrit.

Abdoulaziz KABIROU AMOUSSA: a participé à la collecte et au traitement des données.

Chaibou MAIDAKOUALE: a participé à la collecte des données.

## Liens d'intérêts

Les auteurs déclarent n'avoir aucun conflit d'intérêts.AUTEURS
